# Genetic Dissection of Phosphorus Use Efficiency and Genotype-by-Environment Interaction in Maize

**DOI:** 10.3390/ijms232213943

**Published:** 2022-11-11

**Authors:** Dongdong Li, Guoliang Li, Haoying Wang, Yuhang Guo, Meng Wang, Xiaohuan Lu, Zhiheng Luo, Xintian Zhu, Thea Mi Weiß, Sandra Roller, Shaojiang Chen, Lixing Yuan, Tobias Würschum, Wenxin Liu

**Affiliations:** 1 Key Laboratory of Crop Heterosis and Utilization, The Ministry of Education/Key Laboratory of Crop Genetic Improvement, Beijing Municipality/National Maize Improvement Center/College of Agronomy and Biotechnology, China Agricultural University, Beijing 100193, China; 2 Institute of Plant Breeding, Seed Science and Population Genetics, University of Hohenheim, 70599 Stuttgart, Germany; 3 State Plant Breeding Institute, University of Hohenheim, 70599 Stuttgart, Germany; 4 Key Laboratory of Plant-Soil Interaction, The Ministry of Education, Center for Resources, Environment and Food Security, College of Resources and Environmental Sciences, China Agricultural University, Beijing 100193, China

**Keywords:** maize, phosphorus, macro-environment, genotype-by-environment, genome-wide association study, QTL, genomic prediction

## Abstract

Genotype-by-environment interaction (G-by-E) is a common but potentially problematic phenomenon in plant breeding. In this study, we investigated the genotypic performance and two measures of plasticity on a phenotypic and genetic level by assessing 234 maize doubled haploid lines from six populations for 15 traits in seven macro-environments with a focus on varying soil phosphorus levels. It was found intergenic regions contributed the most to the variation of phenotypic linear plasticity. For 15 traits, 124 and 31 quantitative trait loci (QTL) were identified for genotypic performance and phenotypic plasticity, respectively. Further, some genes associated with phosphorus use efficiency, such as Zm00001eb117170, Zm00001eb258520, and Zm00001eb265410, encode small ubiquitin-like modifier E3 ligase were identified. By significantly testing the main effect and G-by-E effect, 38 main QTL and 17 interaction QTL were identified, respectively, in which MQTL38 contained the gene Zm00001eb374120, and its effect was related to phosphorus concentration in the soil, the lower the concentration, the greater the effect. Differences in the size and sign of the QTL effect in multiple environments could account for G-by-E. At last, the superiority of G-by-E in genomic selection was observed. In summary, our findings will provide theoretical guidance for breeding P-efficient and broadly adaptable varieties.

## 1. Introduction

Maize (*Zea mays* L.) is a globally important multi-purpose crop that can be used for food, feed, and industrial purposes. In the next 30 years, the human population is expected to reach 10 billion [1], and thus, a further increase in crop production is required to meet the demand. Maize also serves as a model crop for genetic research, and the availability of a reference genome has enabled significant progress in the genetic and molecular understanding of traits and characteristics related to breeding [2].

Plants are fixed in their environment and have therefore evolved mechanisms to respond to environmental cues. These responses allow them to react to the biotic and abiotic factors to which they are exposed. The phenotype of an individual is jointly determined by the genotype and the environment as well as by their complex interaction. When genotypes behave differently relative to each other in different environments, this plasticity is referred to as genotype-by-environment interaction (G-by-E). In general, the phenotype of plants in two environments can be categorized into four types: additive, divergence, convergence, and crossover types [3]. Regarding a single gene, for the additive type, the effect size and the sign is the same in all environments, so no interaction exists. For the divergence and convergence types, the effect size of the gene is different, but the sign is the same across environments. For the crossover type, by contrast, the sign of the gene’s effect differs depending on the environment. An approach to quantifying the plasticity of plants is the Finlay–Wilkinson regression [4]. Here, the slope and residual variance parameters from these regressions provide two measures of phenotypic response referred to as linear and non-linear [5] or type II and type III plasticity [6]. Linear plasticity refers to the change in the performance of genotypes from one environment to another relative to the average response for this environmental gradient. Non-linear plasticity refers to the variation of a genotype around this regression line of its performance. The phenotypic plasticity of plants is complex by nature, and previous work on its genetic control has shown it to be a highly quantitative trait [5,6].

Phosphorus (P) is one of the essential elements for plant growth and development, but the non-renewable phosphate rock resources are expected to be exhausted in the next 100–400 years [7]. In addition, P fertilizer use is associated with severe adverse effects on the environment. So far, the most effective way to solve this problem is to breed varieties with a higher P use efficiency, which means developing varieties that can achieve high yields also under reduced P availability. In crops, only a few genes, for example, *GmPHF1* [8], related to P use efficiency, are mined through forward genetics. Generally, in agriculture, the environment or the macro-environment could be the combination of year, location, fertilizer treatment, etc., consisting of predictable and unpredictable environmental factors. For a long time, complex environmental factors were mixed together and could not be separated and quantified, resulting in genetics; we call the interaction between genotype and environment as genotype-by-environment but not genotype-by-specific environmental factors. Recently, the researchers applied several environmental indexes like photothermal time [9], growing degree-days [10], and diurnal temperature range [11] to explore the interaction among genotype, environment, and development. The phosphorus concentration in the soil is one of the important environmental characteristics affecting crop growth. To our knowledge, when treating the P as the environment factor, there is no report to dissect the genotype-by-P in genome-wide association study (GWAS) when exposed to different P treatments in maize, where the effect size and sign of interaction between quantitative trait loci (QTL) and P could be quantified, which is an attractive topic and even for other nutrient elements.

In this study, we assessed a large maize population in seven macro-environments, i.e., year, location, and P treatment combinations, for 15 traits, including flowering time, agronomic and yield traits, in order to phenotypically and genetically dissect the interaction between the genotype and the environment. In particular, the objectives of this study were to assess the genotype-by-environment and to characterize the underlying genetic control as well as to identify genes related to P use efficiency.

## 2. Results

### 2.1. Analysis of Olsen-P in the Soil

**Figure 1:**
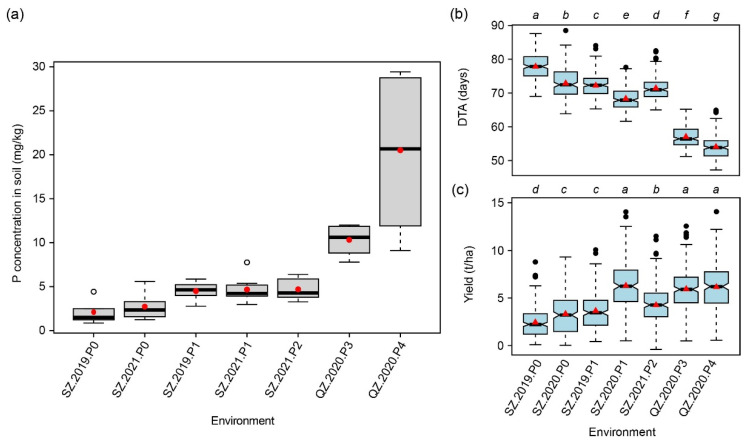
Soil P concentration and trait distributions. (**a**) Soil P concentration of the seven macro-environments, (**b**) distribution of days to anthesis (DTA), and (**c**) yield in these macro-environments. Multiple comparisons with letters were made by the least significant difference method at the significance level of 0.05.

Nine soil samples were taken evenly in the P0 and P1 trials in 2019, and the mean Olsen-P was 2.10 mg/kg and 4.50 mg/kg, respectively (Figure 1a). Nine samples were also taken in the P0, P1, and P2 trials in 2021 and for P3 and P4 in 2020, and the mean Olsen-P was 2.72 mg/kg, 4.67 mg/kg, 4.70 mg/kg, and 10.32 mg/kg and 20.51 mg/kg, respectively. The Olsen-P level in P0 and P1 was similar across the two years, indicating a stable P condition. Based on the P concentration, three different P levels, low-P level (P0), normal-P level (P1 and P2), and high-P level (P3 and P4), can be classified. Due to the substantial differences among the P levels, we considered the year, the location, and the P level to define the macro-environments used in this study.

### 2.2. Population Analysis

**Figure 2:**
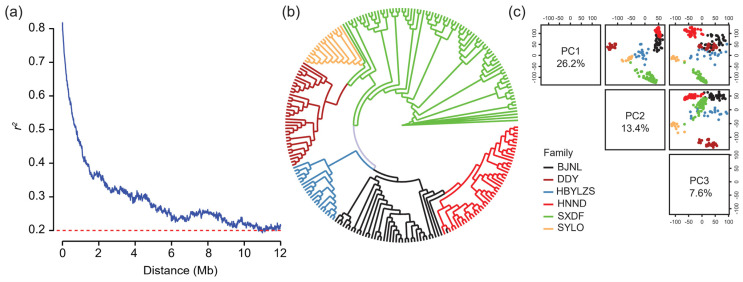
Molecular analysis of the maize population. (**a**) Decay of linkage disequilibrium with physical distance. (**b**) Neighbor-joining tree of the 234 doubled haploid lines from six families and (**c**) principal component analysis plots of the first three main components.

This study is based on 234 doubled haploid (DH) lines derived from six biparental families (Table S1). All lines were sequenced for genotyping with a sequencing depth of ~14X for the parents and ~2X for the progeny (Figure S1), so after quality checks, 136,792 single nucleotide polymorphisms (SNPs) were obtained. These high-quality SNPs were used to molecularly characterize the population. The SNPs were evenly distributed in the genome and linkage disequilibrium decayed with physical distance to *r*^2^ equal to 0.2 after around 11 Mb (Figure 2a). Phylogenetic analysis was consistent with the pedigree information, separating the lines into six families. The female parent of the population BJNL (Jing2481) and the male parent of HNND (Jing24) was selected from the same cross, which resulted in the two families, HNND and BJNL, being more closely related and grouped (Figure 2b). The first three principal components explained 26.2%, 13.4%, and 7.6% of the variance, and the six families were clearly separated by the first two principal components (Figure 2c).

### 2.3. Summary Statistics for 15 Traits Evaluated at Seven Macro-Environments

A large phenotypic variation was observed for all 15 traits (Table 1, Figure S2). Among these traits, the flowering time-related traits, especially DTA, were sensitive to the environment, as significant differences were observed among all macro-environments (Figure 1b). For the high-P levels (P3 and P4), only five traits, including days to silking (DTS), days to heading (DTH), DTA, anthesis-silking interval (ASI), and ear diameter (ED), showed significant phenotypic differences (Figure 1b and Figure S2), but no significant difference was observed for the other traits, like yield, which means that the yield level did not continue to increase with the increase of available P (Figure 1c). Yield showed significant negative correlations with the flowering time-related traits (Figure S3) and had the highest correlations with kernel number per row (KNPR) (*r* = 0.67, *p* < 0.01) and ED (*r* = 0.62, *p* < 0.01).

The genetic variance $\sigma_{g}^{2}$ and the genotype-by-environment interaction variance $\sigma_{ge}^{2}$ were both significant for all traits (Table 1). For the ratio between the two ($\sigma_{ge}^{2}$/$\sigma_{g}^{2}$), KNPR had the largest value with 0.46, followed by yield with 0.42. The heritability was high for all traits, ranging between 0.82 for ASI and 0.95 for plant height (PH) and ear height (EH). In summary, large phenotypic variations, significant genetic and genotype-by-environment interaction variances, and high heritabilities illustrate the high quality of the data, which lays the foundation for genetic and genomic analyses.

### 2.4. Finlay–Wilkinson Regression with Seven Macro-Environments

**Figure 3:**
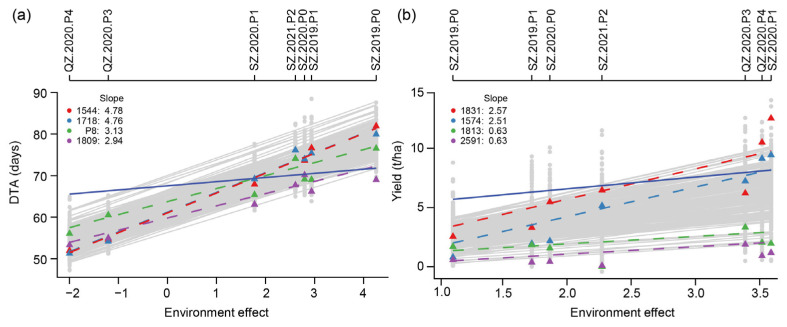
Environment effect and genotype plasticity. Finlay–Wilkinson regression analysis for (**a**) DTA and (**b**) yield. Only the four lines with the two highest and lowest linear plasticity values for the trait are shown. The dashed lines represent the slope (linear plasticity) of individual genotypes; the greater the slope, the greater the linear plasticity of the plant. The solid blue line represents a slope of one.

To characterize the response of the genotypes to the environment, the Finlay–Wilkinson regression was used to calculate the linear and the non-linear plasticity (Figure 3 and Figure S4). For DTA, the largest environmental effect was observed for SZ.2019.P0 with a value over 4, and the smallest value was around −2 for QZ.2020.P4. The linear plasticity (i.e., the slope of each genotype) ranged between 2.94 for the line “1809” and 4.78 for line “1544”, which means that DTA increased by 2.94 or 4.78 per unit increase of the environment effect. So of the two lines, line “1544” showed a stronger response to the environment (Figure 3a). For yield, the environment effect was largest for SZ.2020.P1 with a value of 3.6 and smallest for SZ.2019.P0 with a value of 1.11. Line “1831” had the highest linear plasticity with a value of 2.57, and line “2591” had the lowest linear plasticity with a value of 0.63 (Figure 3b). These variations in linear plasticity were also observed for the other traits (Figure S4). In addition, low correlations were observed between the two plasticity indexes that were only significant (*p* < 0.05) for PH, EH, ELL, RNPE, KNPR, and yield (Table S2). Furthermore, for most of the traits, the non-linear plasticity showed a larger variation, as measured by the quartile coefficient of dispersion, which means that the response varied among genotypes, highlighting the complexity of the genotype-by-environment interaction and different genetic architectures of the two plasticity indexes.

### 2.5. Contribution of Different Gene Regions to the Linear and Non-Linear Plasticity

**Figure 4:**
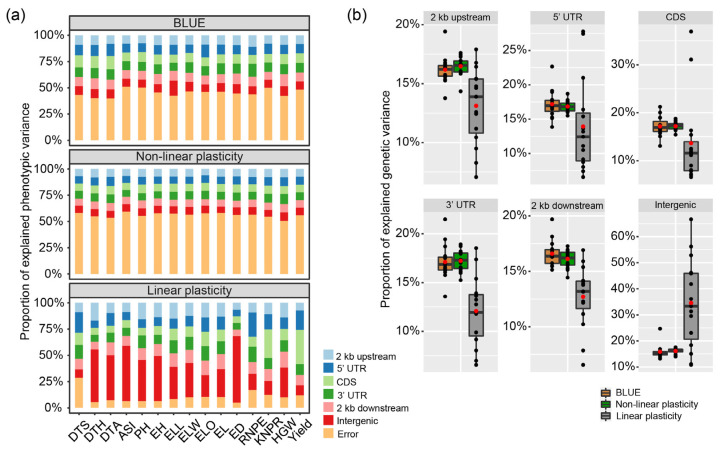
Contribution of different gene regions to genotypic performance and plasticity. Proportion of (**a**) phenotypic and (**b**) genetic variance explained by SNPs in six categories: 2 kb upstream, 5′ UTR, CDS, 3′ UTR, 2 kb downstream and intergenic, shown for the BLUE across seven environments, the non-linear plasticity, and the linear plasticity. DTS, days to silking; DTH, days to heading; DTA, days to anthesis; ASI, anthesis-silking interval; PH, plant height; EH, ear height; ELL, ear leaf length; ELW, ear leaf width; ELO, ear leaf order; EL, ear length; ED, ear diameter; RNPE, row number per ear; KNPR, kernel number per row; HGW, hundred-grain weight; Yield, yield per hectare. The black point indicate each value of trait. The red points indicate mean values.

The whole-genome SNPs were assigned to six gene region categories, i.e., 2 kb upstream of a gene, 5′ untranslated regions (5′ UTR), coding sequence (CDS), 3′ untranslated regions (3′ UTR), 2 kb downstream of a gene and intergenic regions, which resulted in 4959, 2059, 11,382, 2180, 4814, and 95,303 SNPs, respectively. The six marker datasets were then used to calculate the contribution of the different gene regions to the explained variance of the genotypic performance or called the best linear unbiased estimator (BLUE), the linear and the non-linear plasticity (Figure 4a). For the BLUE, the proportion of the total genetic variance, i.e., the proportion of the phenotypic variance that is not an error, ranged for the 15 traits between 49.2% for ASI and 60.3% for DTA. For the non-linear plasticity, it ranged between 40.7% for ASI and 49.6% for HGW, and for the linear plasticity, it ranged from 71.4% for DTS to 95.2% for ED. Generally, the proportion of explained genetic variance was highest for the linear plasticity, followed by the BLUE and the non-linear plasticity. Next, we investigated whether these six gene categories contribute equally to the total genetic variation (Figure 4b) and found this to vary for the BLUE, the linear plasticity, and the non-linear plasticity, as well as among the 15 traits. It showed that SNPs in the intergenic region contributed the most to the variations of two plasticity indexes across 15 traits. Notably, there is also variation among traits; for example, for the linear plasticity, the SNPs in the 5′ UTR region explained a proportion of the genetic variance of 27.7% for RNPE compared to the average of 13.9%, the SNPs in the CDS explained 36.9% for yield, while the average was only13.6%, and SNPs in the intergenic region explained 56.2% for ASI and 66.7% for ED, respectively. In summary, the obtained results highlight the important role of variation outside the coding regions for performance and plasticity, particularly for the linear plasticity in putative regulatory elements located in intergenic regions.

### 2.6. Genome-Wide Association Mapping for Genotypic Performance, Linear Plasticity, and Non-Linear Plasticity

**Figure 5:**
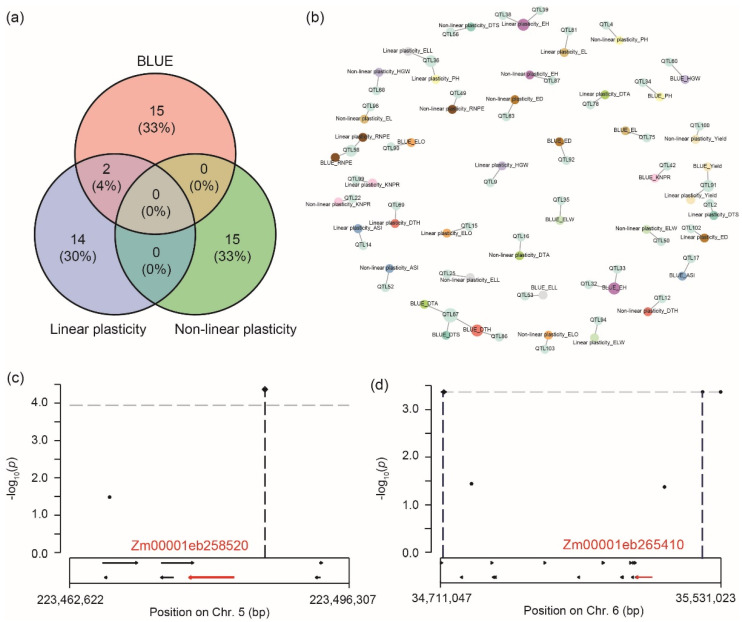
QTL mapping for 15 traits for the genotypic performance (BLUE) across seven environments, the non-linear plasticity, and the linear plasticity. (**a**) Venn diagram showing overlapping and specific QTL. (**b**) Trait-QTL network. The different colors represent different traits and QTL. (**c**) Identification of the gene Zm00001eb258520 as a candidate for QTL64 (chromosome 5: 223.44–223.64 Mb) detected for RNPE in the P-stress environment SZ.2019.P0. (**d**) Identification of the gene Zm00001eb265410 as a candidate for QTL65 (chromosome 6: 30.75–37.44 Mb) detected for DTS in the P-stress environment SZ.2019.P0. DTS, days to silking; DTH, days to heading; DTA, days to anthesis; ASI, anthesis-silking interval; PH, plant height; EH, ear height; ELL, ear leaf length; ELW, ear leaf width; ELO, ear leaf order; EL, ear length; ED, ear diameter; RNPE, row number per ear; KNPR, kernel number per row; HGW, hundred-grain weight; Yield, yield per hectare.

As a first step, cob color as a trait with a simple genetic architecture and a high heritability was used for genome-wide association mapping. This identified the causal gene *P1* (*pericarp color1*) [12] in the QTL region on chromosome 1, illustrating the QTL detection power and the mapping resolution that can be achieved in this population (Figure S5). We then performed genome-wide association mapping for the genotypic performance (BLUE) in each environment, the BLUEs across environments, the linear plasticity, and the non-linear plasticity values, which identified 107, 17, 16, and 15 QTL, respectively (Table S3). No overlapping QTLs were identified between the BLUE across environments and the non-linear plasticity or between the two plasticity measures, and only two overlapping QTLs were observed between the BLUE and the linear plasticity (Figure 5a). These two QTLs were QTL58 (chromosome 5: 211.05–212.48 Mb), identified for RNPE, and QTL91 (chromosome 8: 177.89–178.59 Mb), identified for yield. Regarding the 15 traits, many pleiotropic QTL were found (Figure 5b), for example, QTL67 (chromosome 6: 111.67–112.85 Mb) with a pleiotropic effect on DTA, DTS, and DTH, or QTL36 (chromosome 4: 28.84–28.93 Mb) that was significantly associated with the linear plasticity of PH and ELL.

In addition, association mapping was also performed for the genotypic performance within each environment, and candidate QTL was used for further mapping (Table S3). In the environment, SZ.2021.P2, QTL28 (chromosome 3: 1.72–2.01 Mb) was identified for DTS, DTH, and DTA, and the QTL region harbors a gene (Zm00001eb119170, chromosome 3: 1,892,283–1,901,302 bp) that was also a candidate gene in a previous reported [13]. For yield, QTL91 (chromosome 8: 177.89–178.59 Mb) was one of the two overlapping QTL between BLUE across environments and linear plasticity, and in this region, a candidate gene (Zm00001eb369550, chromosome 8: 178,138,558–178,139,195 bp) is located that was reported for grain yield in a maize multiparent advanced generation intercross population [14].

In the low-P stress environment SZ.2019.P0, a QTL for ASI and DTH was detected (QTL26, chromosome 2: 239.61–240.63 Mb), and a candidate gene (Zm00001eb117170, chromosome 2: 240,121,115–240,126,202 bp) for this QTL was identified that was recently reported for the number of root forks and root tip number in a maize association panel [15]. QTL64 (chromosome 5: 223.44–223.64 Mb) was detected for RNPE, and the QTL region harbors the gene Zm00001eb258520 (chromosome 5: 223,477,062–223,482,483 bp), encoding phosphate transporter 3 protein, that plays an important role in Pi acquisition, transport and allocation (Figure 5c) [16,17]. In the same low-P environment, QTL65 (chromosome 6: 30.75–37.44 Mb) was identified for DTS, for which a novel putative candidate gene (Zm00001eb265410, chromosome 6: 35,283,172–35,331,023 bp) could be identified (Figure 5d). The orthologous gene in *Arabidopsis* is AT5G60410 (*ATSIZ1*), which encodes a small ubiquitin-like modifier E3 ligase, known to be involved in the control of Pi starvation-dependent responses [18].

### 2.7. Genome-Wide Association Mapping for Genotype-by-Environment Interactions

**Figure 6:**
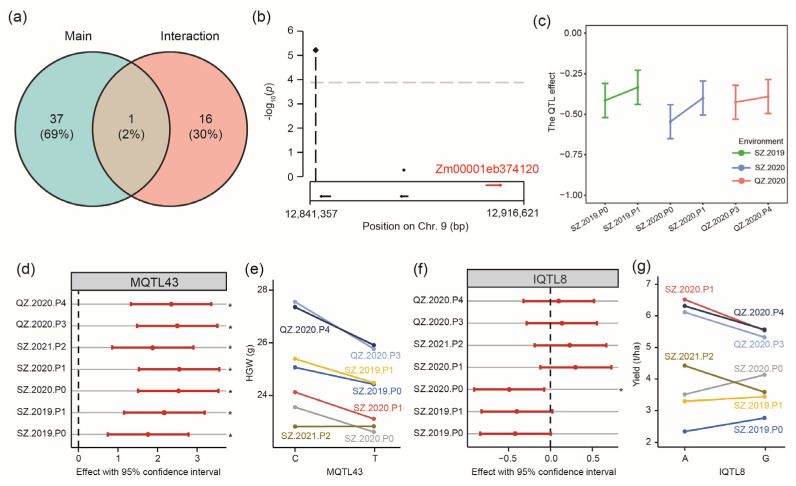
QTL mapping for main and locus-by-environment interactions. (**a**) Venn diagram of main QTL and G-by-E QTL for 15 traits. (**b**) Identification of the gene Zm00001eb374120 as main QTL MQTL38 (chromosome 9: 12.46–13.06 Mb) for ELW. (**c**) The effect of MQTL38 in paired environments. (**d**) Genotype × environment interaction effects (GGE)-type marker effect for main QTL MQTL43 (chromosome 9: 121.45–121.46 Mb) identified for hundred-grain weight (HGW). (**e**) Mean HGW of the two QTL alleles in the seven environments. (**f**) GGE-type marker effect for the interaction QTL IQTL8 (chromosome 2: ~214.58 Mb) identified for yield and (**g**) mean yield distribution of the two QTL alleles in the seven environments. The significance of GGE-type effects was tested by the Wald test, * means the effect is significant at the level of 0.05.

To explore the genetic control underlying the genotype-by-environment interaction, we performed genome-wide association mapping for the main marker effect that was constant across environments and for genotype-by-environment interactions that were specific to the environment (Table S4). For the 15 traits, 38 main effect QTL and 17 genotype-by-environment interaction QTL were identified. Only one QTL (MQTL27, chromosome 2: 118.71–119.63 Mb) related to EH was found to have both a main and a genotype-by-environment interaction effect (Figure 6a, Table S4).

We again aimed to identify candidate genes by mapping and to further characterize the QTL and their response to the environment. MQTL38 (chromosome 9: 12.46–13.06 Mb) has the main effect on ELW (Figure 6b, Table S4), and within this QTL interval, the candidate gene Zm00001eb374120 (chromosome 9:12,903,174–12,908,621 bp) is located, that encodes a Pi starvation-induced transcription factor whose overexpression could improve the low-P tolerance of maize [19]. More importantly, the interaction effects of MQTL38 with the favorable allele ‘AA’ were significant (*p* < 0.05) across all environments and had the same sign, meaning that the QTL was stable and thus is of potential value in plant breeding (Figure 6c). Another example of a persistence type QTL is MQTL43 (chromosome 9: 121.45–121.46 Mb), a QTL with a significant main effect identified for HGW. The sign of the QTL effect was consistent across environments, meaning that the allele ‘CC’ was the favorable allele in each of the seven environments (Figure 6d,e and Table S5).

Regarding genotype-by-environment interaction QTL, most of them belong to the crossover type, the most problematic type in plant breeding, as this means that the effect and its sign vary with the environment (Table S5). For example, for IQTL8 (chromosome 2: ~214.58 Mb) identified for yield, positive QTL effects were found for ‘AA’ in four environments, whereas the favorable effect came from ‘GG’ in the other three environments (Figure 6f,g and Table S5). The inconsistency of the effects and signs of QTL across environments illustrates the genotype-by-environment interaction at the molecular level. In this case, selection of the one favorable allele in plant breeding is not possible because the favorable allele changes with the environment. Thus, a better characterization of such loci is required in order to assist breeding and to allow the selection of the alleles most suitable for the intended target environment.

### 2.8. Genomic Prediction for Genotype-by-Environment Interaction

**Figure 7:**
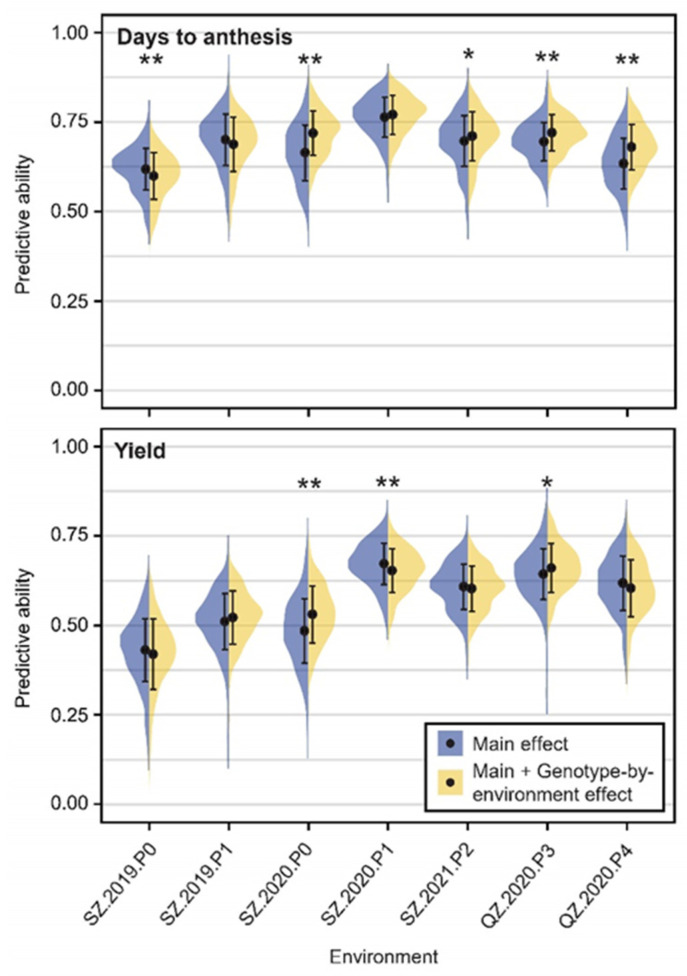
Genome-wide prediction results. Predictive ability in each environment using a model with only marker main effects or integrating main and genotype-by-environment interaction effects shown for days to anthesis and yield per hectare. The significance test between the two models was performed by *t* test. *, significant at 0.05 level; **, significant at 0.01 level. The point and bar represent mean and standard deviation value, respectively.

To evaluate the potential of genomic prediction for crop improvement, two prediction models integrating only marker main effects or main and genotype-by-environment interaction effects were implemented and tested for the 15 traits (Figure 7, Table S6). Generally, EH yielded the highest and ASI the lowest predictive ability across seven environments, which is consistent with the highest and lowest heritability, respectively (Table 1). For DTA, in four of five environments with a significant difference, the model incorporating the genotype-by-environment effect achieved a higher predictive ability than the model without (Figure 7). For yield, this was observed for two of three environments with a significant model difference (Figure 7). In conclusion, the integration of multi-environment data is promising for genomic prediction and provides a strategy for utilizing genotype-by-environment interactions in molecular breeding.

## 3. Discussion

### 3.1. The Macro-Environment in Plant Breeding

The yield of crops realized in the field is determined by the genetic potential of the variety and the environment, as well as by their complex interaction. Especially with the effects of climate change and the resulting increasing occurrence of extreme weather conditions, it is crucial to better understand the interaction of crops with the environment. Generally, the external abiotic and biotic factors that affect the growth of crops are defined as the environment [20], which could also be called a macro-environment. In contrast, the micro-environment, defined as the growth environment of a single plant or a plot, cannot be parameterized and is usually regarded as an error [21]. In our study, we combined year, site, and P availability as macro-environments, thereby focusing on the P level (Figure 1a). This revealed substantial phenotypic variation within as well as among macro-environments (Figure 1 and Figure S2).

In breeding, the macro-environment is often treated as just a few factors: year, location, treatment, and their interactions. However, the macro-environment contains many vital meteorological factors, such as precipitation, temperature, or solar radiation, but also agronomic factors like the amount of fertilizer applied. Deciphering and quantifying the impact of environmental factors on crop growth is a concept proposed as envirotyping [22]. In this way, the genotype-by-environment interaction, which is highly multifactorial and, therefore, a complex black box, can be decomposed into the interaction between the genotype and different environmental factors. In human genetic studies, the environment was refined into 60 lifestyles, such as habits and exercise, and an efficient computational model was developed that allows to identify loci underlying the genotype-by-environment interaction [23]. Thus, future work should also focus on refining the environment into its components toward a better understanding of QTL and how they interact with the different environmental parameters.

### 3.2. How to Interpret and Utilize Genotype-by-Environment Interactions

Genotype-by-environment interaction describes the non-parallel performance of genotypes across environments, which can potentially be problematic in plant breeding. The performance of crops in different environments can be categorized as an additive, divergence, convergence, and crossover type [3]. From a plant breeding point of view, for the first three types, the performance of the material can be judged in a few representative environments since the ranking does not change among them. For the fourth type, however, the performance must be judged in all target environments to identify superior genotypes under the respective conditions. Our study also provided a molecular explanation for this type of interaction, as our results showed that QTL effects change in size and sign depending on the environment (Figure 6, Table S5). High and stable performance of varieties, especially for yield, is the main goal in breeding. To achieve this, selection must combine favorable alleles, but attention should also be paid to the consistency of QTL effects across environments. An example of this is the crossover type QTL IQTL8 identified here (Figure 6f,g), for which the QTL effect sign was different in three environments compared to the other four environments. In such cases, alternative alleles could be selected that show little or little dependency on the environment. Alternatively, if this is not possible, different genotypes are required for the different mega-environments and their prevailing climatic conditions. In practice, commercial maize varieties are hybrids, not lines, as investigated here, and the hybrids’ performance is controlled by additive, dominance, and epistatic genetic effect [24]. Consequently, the genotype-by-environment interaction system is more complex, including additive-by-environment, dominance-by-environment, and epistatic-by-environment interactions. Future research is required to investigate the genotype-by-environment interaction and its genetic control in a hybrid background.

### 3.3. Contribution of Different Gene Regions to Genotypic Performance and Plasticity

In order to better understand the mechanisms underlying genotypic performance and plasticity, we evaluated the global contribution of SNPs in different genomic regions. To this end, we assigned SNPs to six datasets based on their relative physical position in or around genes and found that these six categories contributed unequally to the phenotypic and genetic variation of the mean performance and plasticity of the traits (Figure 4). In a previous study, Kusmec et al. (2017) demonstrated that SNPs associated with the mean phenotype were significantly enriched in the exons and 5 kb upstream regions of genes. The upstream region of genes typically corresponds to the promoter, harboring the genes’ regulatory sequences. SNPs significantly associated with linear plasticity were only significantly enriched in the exon regions, and for the non-linear plasticity, there was no significant enrichment in any region. In another study, SNPs associated with the slope were significantly enriched in the regions upstream of genes and significantly decreased within genes, while SNPs significantly associated with the per se performance were not significantly enriched in any of the gene regions [6]. In our study, we found that the intergenic regions contributed more to the linear plasticity than the other regions, suggesting the presence of regulatory elements in these intergenic regions (Figure 4). The reasons for the different results obtained in different studies may be the differences in the study material, population type, test environments, or gene structure annotation. We also observed low correlations, and no overlapping QTL between linear and non-linear plasticity, indicating different genetic architectures and genomic regulation. Our results thus provide further support for the hypothesis that the variation in gene regulatory elements plays a key role in plant plasticity. We observed this, especially for ED and the adaptation-related trait DTH, which is in line with results from other studies that highlighted the function of intergenic regions for regulatory and biological processes [25,26]. Thus, for the process of plants adapting to environmental changes, be it in agriculture or in natural settings, the regulatory region may be more flexible and thus better suited to generate novel variation than alterations in the gene sequence.

### 3.4. Mine Gene with Genotype-by-P Treatment

In agriculture, the environment is a general concept and includes many predictable and unpredictable factors. In this study, we divided these factors into P treatments and the others by setting different P treatments in the trials. To mine genetic factors that turely associated with phosphorus, the following strategies were applied. Firstly, GWAS was performed for genotypic performance in each environment and phenotypic plasticity. In single environments, specifically in P deficiency environments, QTL and underlying candidate genes were identified, for example, QTL64 with the candidate gene Zm00001eb258520 or QTL65 with the candidate gene Zm00001eb265410. Secondly, by taking all macro-environments into one association mapping analysis, QTL for genetic and interaction effects with the macro-environment were identified. In this study, the macro-environment included main environments and P treatments. So, biological interpretation was needed to separate QTL with G-by-E and G-by-P in the GWAS for interaction. Subsequent characterization revealed candidate genes related to P-stress response, for example, MQTL38 with the candidate gene Zm00001eb374120 (Figure 6b). In seven environments, its effects differed significantly from 0, and the signs were the same, which meant that the QTL had a consistent effect in environments (Table S5). Interestingly, its effect (the absolute value) in SZ.2019.P0 was larger than SZ.2019.P1, its effect in SZ.2020.P0 was larger than SZ.2020.P1, and its effect in QZ.2020.P3 was larger than QZ.2020.P4 (Figure 6c). In summary, in the same year, the QTL effect was always larger in relatively P-deficient environments. So, we could infer that the QTL effect may vary with the density of P stress. A previous report about this gene showed the expression in the root increased with the low-P treatment time but did not accrue in stem and leaf [19], which was consistent with our hypothesis and showed that the gene played an important role in root development further affected the traits of shoot and leaf in P deficiency environments.

## 4. Materials and Methods

### 4.1. Materials

The maize material used in this study consisted of a total of 234 lines in six DH populations developed from different pedigrees (Table S1), where the parents are elite lines of hybrid varieties in different breeding institutes in China. The populations are named SYLO, HBYLZS, DDY, BJNL, HNND, and SXDF and have sizes of 17, 22, 35, 38, 44, and 78 genotypes, respectively.

These individuals were evaluated under P0 and P1 treatments at the China Agricultural University Shangzhuang Station (SZ) (40.14° N, 116.19° E) in Beijing in 2019 and 2020; the macro-environments were called SZ.2019.P0, SZ.2019.P1, SZ.2020.P0, and SZ.2020.P1, respectively. The P0 and P1 treatments were reported before [17]. In brief, for P0, no phosphorus fertilizer was used since 1985, and for P1, 45 kg/ha P_2_O_5_ was applied before sowing. In addition, traits were recorded in another P treatment with 90 kg/ha P_2_O_5_ in 2021 at this location, and the environment was called SZ.2021.P2. For P0, P1, and P2, 240 kg/ha N fertilizer was applied. Further agronomic managements were identical for all three P treatments. Traits were recorded in the P3 and P4 treatments in 2020 at the China Agricultural University Quzhou Station (QZ) (36.86° N, 115.02° E), Hebei province. For P3, no phosphorus fertilizer was used from 2018 to 2020, and for P4, 45 kg/ha P_2_O_5_ was added. Both trials received an equal amount of 150 kg/ha N fertilizer. The macro-environments were named QZ.2020.P3 and QZ.2020.P4, respectively. The Olsen-P was measured following a standard method [27].

In each environment, an augmented alpha design with two replicates was used. Blocks were nested in each replicate. In each block, two standard checks, Zheng58 and Chang7-2, two elite inbred lines in China, were included. The row length was 1.6 m (SZ.2020.P0, SZ.2020.P1, SZ.2019.P0, SZ.2019.P1) and 2 m (SZ.2021.P2, QZ.2020.P3, QZ.2020.P4), the plant distance 0.2 m and the row distance 0.5 m. According to previous study [17,28,29], different P treatments could influence the performance in development stages, plant architecture, agronomic traits, and yield-related traits. A total of 15 traits were recorded based on described methods [28,30], including flowering time-related traits with DTS, DTH, DTA, and ASI; agronomic traits including, PH, EH, ELL, ELW, and ELO; and yield-related traits including EL, ED, RNPE, KNPR, HGW, and Yield.

### 4.2. Phenotypic Analysis

Two linear models were applied to calculate the BLUE value, the variance components, and the heritability.

In each environment, the linear mixed model was:

(1) $$y_{im}=\mu +G_{i}+R_{m}+B_{n}(R_{m})+\varepsilon_{im},\;$$

where $y_{im}$ is the phenotype of *i*th line in *m*th replicate, μ the average, $G_{i}$ the genetic effect of the *i*th line, $R_{m}$ the effect of the *m*th replicate, $B_{n}$ the effect of the *n*th block in the *m*th replicate, and $\varepsilon_{im}$ the error following a heterogeneous distribution $\varepsilon_{im}\sim N(0,\sigma_{m}^{2})$, with $\sigma_{m}^{2}$ being the variance of error in the *m*th replicate.

To analyze the genetic and genotype-by-environment interaction, the following linear model was used:

(2) $$y_{ijm}=\mu +G_{i}+E_{j}+G*E_{ij}+R_{m}(E_{j})+B_{n}(E_{j}R_{m})+\varepsilon_{i}{}_{jm},$$

where $y_{ijm}$ is the phenotype of the *i*th line in the *j*th environment and the *m*th replicate, μ is the average, $G_{i}$ the genetic effect of the *i*th line, $E_{j}$ the effect of the *j*th environment, $G*E_{ij}$ the interaction of the genetic effect of the *i*th line and the *j*th environment, $R_{m}(E_{j})$ the effect of the *m*th replicate in the *j*th environment, $B_{n}(E_{j}R_{m})$ the effect of *n*th block in the *m*th replicate in the *j*th environment, and $\varepsilon_{ijm}$ the error following a heterogeneous distribution $\varepsilon_{i}{}_{jm}\sim N(0,\sigma_{j}^{2})$, with $\sigma_{j}^{2}$ being the variance of error in the *j*th environment.

First, the Studentized Residual Razor method was used to remove outliers in the second linear model with a threshold of 2.8 [31]. Then, the above two models were used to calculate the BLUE with the genotype modeled as fixed effect and the heritability with all the variables as random. Cullis’ formula was used to calculate the broad-sense heritability [32]. All mixed model calculations were performed with ASReml-R (version 4.1, UK) [33].

### 4.3. Genotyping and Quality Control

At the three- or four-leaf stage, leaves were taken for DNA extraction using a cetyltrimethylammonium bromide method [34]. After quality control of the DNA, at least 1.5 μg DNA was broken into fragments of 350 bp and then used to construct sequencing libraries that were sequenced on an Illumina HiSeq 2000 system. Reads with N content over 10% and low-quality (Q ≤ 5) over 50% were discarded. The remaining reads were aligned to the reference genome Zea_mays.B73_RefGen_v4.dna.toplevel.fa using BWA (version 0.7) [35]. The software SAMtools (version 1.6) was used to sort the mapped reads and filter for duplicates as well as for SNP calling [36]. The SNP genotype dataset of each of the six populations was extracted from the whole VCF files using vcftools (version 0.1.17) [37], and loci with mapping quality below 5, missing rate over 0.3, minor allele frequency (MAF) below 0.05, and heterozygous rate over 0.3 were filtered out. Then, the heterozygous loci were set to missing values using Tassel (version 5.2.78) [38], and loci showing segregation distortion (*p* < 0.005) were filtered by following the expected ratios of 3:1 for the BC_1_DH SXDF population and 1:1 for the remaining F_1_DH populations. Last, Beagle (version 5.2) was used for imputation using the default parameters [39].

Then a sliding window method described before [40] was used to correct potential genotyping errors and find the breakpoints using the following steps: (1) the raw SNPs were scanned with a 15-SNPs window and a sliding step of one SNP. In each window, the genotype was determined by the ratio of the two parents. If the ratio of P1:P2 was over 11:4, the window was determined to be the P1 genotype and vice versa. Heterozygous genotype was determined when the ratio of P1:P2 was between 4:11 and 11:4. In each window, the SNP genotypes inconsistent with the window genotype were corrected. The breakpoint was determined when the homozygous P1 genotype was changed into homozygous P2 or vice versa. The above process was performed in each bi-parental family. As a result, the breakpoints were obtained for each line. The fragments between two breakpoints were called bins [41]. As some loci were not polymorphic across the six populations, when all polymorphic loci were merged, non-polymorphic loci in a family were treated as missing genotypes. Based on the breakpoint information, a strategy to project the parents’ genotype with high coverage onto the progeny was used to impute the rest of the missing genotype [42]. The core principle of such a projection is that the genotype of progeny in a bin should have the same genotype as the parents.

The physical position in the B73_RefGen_v4 was converted to Zm-B73-REFERENCE-NAM-5.0 in Ensembl Plants using the function “Assembly Converter” by choosing “B73_RefGen_v4→Zm-B73-REFERENCE-NAM-5.0”. Then the genotype of ATCG was transformed into a numeric format based on the allele frequency; the allele with a higher frequency becomes ‘1′, and the minor allele becomes ‘−1′.

### 4.4. Population Analysis

The high-quality SNPs were used for the population analyses, including phylogenetic tree, principal component analysis (PCA), and linkage disequilibrium (LD) analysis. The function “Distance Matrix” in Tassel (version 5.2.78) [38] was used to calculate the identity by state distance matrix. The approach “Neighbor Joining” was used to construct the polygenetic tree, and visualization was performed by the R package ggtree [43]. The “PCA” module in Tassel was used to calculate the PCA, and the component number was set to six. The LD decay with distance was calculated in PopLDdecay (version v3.40Beta) [44] with -MaxDist 12,000 and the other parameters as default.

### 4.5. Finlay–Wilkinson Regression Analysis

In multi-environment trials, the sensitivities of an individual to the environment can be parameterized by Finlay–Wilkinson regression [4]. The formula was:

(3) $$y_{ij}=\mu +g_{i}+(1+b_{i})h_{j}+\varepsilon_{i}{}_{j},\;$$

where $y_{ij}$ is the BLUE value of the *i*th line in the *j*th environment, $g_{i}$ is the effect of the *i*th line following a distribution $g\sim N(0,K\sigma_{g}^{2})$, $h_{j}$ is the effect of the *j*th environment following a distribution $h\sim N(0,H\sigma_{h}^{2})$, and $\varepsilon_{ij}$ the error following a distribution $\varepsilon \sim N(0,I\sigma^{2})$. The term $\left(1+b_{i}\right)$ can be interpreted as the linear plasticity of the *i*th line to the environment [5], where $b\sim N(0,K\sigma_{b}^{2})$. The variance of $\varepsilon_{i}{}_{j}$ for each line in all environments can be interpreted as non-linear plasticity [5]. The variance was log-transformed. The interpretation is that a line with $\left(1+b_{i}\right)$ = 0 has no response to the environment, whereas $\left(1+b_{i}\right)$ = 1 means that a line has average sensitivity. In this study, the kinship matrix *K* was calculated by Xu’s method [45], and *H* was an identity matrix. The above model was solved in the R package FW [46] using a Bayesian method with the parameters nIter 20,000 and burnIn 10,000, and the other parameters as default.

### 4.6. Genome-Wide Association Analysis

We used all the high-quality SNPs (MAF > 0.05) to perform genome-wide association mapping for the 15 traits. The classical linear mixed model (Q + K) was used for GWAS [47], which was achieved in the R package GAPIT (version 3.0) [48] with the principal component number set to six.

For GWAS of G-by-E, the linear mixed model was:

(4) $$y=X\beta +Z_{k}\gamma_{k}+W_{k}\delta_{k}+u+u_{GE}+\varepsilon ,$$

where y was a column vector of the BLUEs in each of the seven environments, β was the fixed effects, and X the respective design matrix including environment indicator and the first six principal components. The total effect of the *k*th marker was decomposed into a constant main effect $\gamma_{k}$ plus a G-by-E effect $\delta_{k}$ specific to the single environments. In the model, the part “Kinship” consists of two parts: one is the main polygenic effect u following a distribution $u\sim N(0,K_{g}\sigma_{g}^{2})$, and the other is the G-by-E polygenic effect $u_{GE}$ following a distribution $u_{GE}\sim N(0,K_{GE}\sigma_{GE}^{2})$. The calculation of the kinship matrices $K_{g}$, $K_{GE}$ and the design matrices $Z_{k}$, $W_{k}$ were described recently [49]. In this study, both model variances and error variance were assumed to be homogenous and were calculated using the restricted maximum likelihood in the R package gaston. The model and significance test for γ and δ was performed in the R package gwasQxE [49]. Then, the genotype main effects and GGE and variance of individual QTL were obtained from the GGE model [49]. The GGE-type interaction effect in each environment was tested using the Wald test following a chi-square distribution with one degree of freedom. The GWAS significance threshold was determined by the false discovery rate at a significance level of 0.05 [50,51].

### 4.7. Confidence Intervals of QTL and Candidate Gene Identification

After performing the GWAS, the following steps were applied to obtain the confidence intervals for the QTL: (1) The SNPs that passed the significance threshold were selected; (2) within 11 Mb (the physical distance after which LD decayed below *r*^2^ = 0.2), the most significant SNP was chosen as lead SNP; (3) the LD (*r*^2^) between the lead SNP and others SNP within the 11 Mb interval was calculated; (4) the SNPs that had *r*^2^ below 0.2 with the lead SNP in the left and right direction were declared as the boundary of the QTL region. The LD degree *r*^2^ was calculated in the R package SNPRelate [52]. The start and end positions of all genes in the gene annotation file (Zea_mays.Zm-B73-REFERENCE-NAM-5.0.51.gff3) were extracted. Then the findOverlaps function in the R package GenomicRanges [53] was used to find candidate genes that overlapped with the confidence intervals of the QTL. In this study, any two QTLs for which the confidence intervals overlapped or the peak position was within 1 Mb were regarded as pleiotropic QTLs. In addition, the candidate genes were searched in the GWAS Atlas database [54] (https://ngdc.cncb.ac.cn/gwas/, accessed on 20 February 2022) to check if the genes were published in former studies. The homologous genes of maize in *Arabidopsis thaliana* and *Oryza sativa* Japonica were identified from the Ensembl Plants database using the R package biomaRt [55,56]. The corresponding genome versions were Zm-B73-REFERENCE-NAM-5.0, TAIR10, and IRGSP-1.0, respectively.

### 4.8. Variance Component Estimation for SNPs in Different Regions

SNPs were assigned to one of the following categories depending on their position relative to the annotated genes: 2 kb upstream, in the coding sequence (CDS), in the 5′ untranslated regions (5′ UTR), in the 3′ untranslated regions (3′ UTR), 2 kb downstream or in the intergenic. Then, a linear mixed model was established as follows:

(5) $$y=X\beta +\xi_{\mathrm{upstream}}+\xi_{5^{\prime }\mathrm{UTR}}+\xi_{\mathrm{CDS}}+\xi_{3^{\prime }\mathrm{UTR}}+\xi_{\mathrm{downstream}}+\xi_{\mathrm{intergenic}}+\varepsilon ,$$

where *y* was the phenotype (BLUE, linear plasticity or non-linear plasticity), X was the indicator for the DH population, $\xi_{\mathrm{upstream}}$, $\xi_{5^{\prime }\mathrm{UTR}}$, $\xi_{\mathrm{CDS}}$, $\xi_{3^{\prime }\mathrm{UTR}}$, $\xi_{\mathrm{downstream}}$ and $\xi_{\mathrm{intergenic}}$ were the polygenic background effects that corresponded to different SNP region categories. These six variables were treated as random and kinship matrixes were calculated following a method developed by Xu [45]. This model was solved by a Bayesian Generalized Linear Regression in the R package BGLR [57] with the settings nIter 20,000 and burnIn 10,000 and the other parameter as default.

### 4.9. Genomic Prediction of Genotype-by-Environment Interaction

The model for prediction of the G-by-E was:

(6) $$y=X\beta +u+u_{GE}+\varepsilon ,$$

where the definitions of all terms were the same as in the above GWAS model (4), except that X included the environment indicators and population indicators. When the term $u_{GE}$ is removed, the prediction model was called main effect model. For cross-validation, in each environment, 70% of the lines were randomly assigned to the training set, and the rest formed the test set. When considering all environments, a cross-validation strategy 2 [58] that was designed to predict materials that have not been evaluated in any trials was used to assess the predictive ability of the model calculated by the correlation between observed and predicted values. We repeated the training set—test set partitioning 200 times to obtain the mean predictive ability. The above model was fitted in BGLR [57] with nIter 20,000 and burnIn 10,000 and the other parameter as default.

## 5. Conclusions

It was found that intergenic regions contributed the most to linear plasticity. By significantly testing the main effect and G-by-E effect, 38 main QTL and 17 interaction QTL were identified, in which MQTL38 contained the gene Zm00001eb374120 related to phosphorus use efficiency, and its effect size and effect sign could account for genotype-by-P treatment, which provided theoretical guidance for molecular breeding of P-efficient, high and stable yield maize varieties.

## Figures and Tables

**Table 1:** Summary statistics for 15 traits in seven environments.

Trait	Min	Max	Mean	$\sigma_{g}^{2}$	$\sigma_{ge}^{2}$	Ratio	***H^2^***
DTS	63.83	78.67	69.99	7.58 **	1.61 **	0.21	0.91
DTH	59.45	73.98	65.09	6.68 **	1.32 **	0.20	0.92
DTA	62.12	76.51	67.76	6.54 **	1.51 **	0.23	0.91
ASI	0.19	6.56	2.14	1.13 **	0.45 **	0.40	0.82
PH	129.25	238.54	184.88	369.22 **	56.22 **	0.15	0.95
EH	30.84	103.68	65.35	145.78 **	24.58 **	0.17	0.95
ELL	55.54	84.90	69.85	29.78 **	6.42 **	0.22	0.93
ELW	5.75	9.39	7.72	0.51 **	0.11 **	0.21	0.93
ELO	5.01	7.65	6.29	0.24 **	0.05 **	0.21	0.93
EL	9.21	15.81	12.42	2.08 **	0.65 **	0.32	0.88
ED	30.37	43.55	36.92	7.31 **	3.08 **	0.42	0.85
RNPE	10.48	18.04	13.64	1.66 **	0.41 **	0.25	0.90
KNPR	12.13	30.13	21.14	11.17 **	5.13 **	0.46	0.85
HGW	16.99	34.22	24.75	9.35 **	2.52 **	0.27	0.90
Yield	1.46	8.15	4.61	1.78 **	0.75 **	0.42	0.83

Note: $\sigma_{g}^{2}$, genotypic variance; $\sigma_{ge}^{2}$, genotype-by-environment interaction variance; Ratio refers to the ratio between $\sigma_{ge}^{2}$ and $\sigma_{g}^{2}$; *H^2^*, broad-sense heritability of the trait. DTS, days to silking (days); DTH, days to heading (days); DTA, days to anthesis (days); ASI, anthesis-silking interval (days); PH, plant height (cm); EH, ear height (cm); ELL, ear leaf length (cm); ELW, ear leaf width (cm); ELO, ear leaf order (count); EL, ear length (cm); ED, ear diameter (cm); RNPE, row number per ear (count); KNPR, kernel number per row (count); HGW, hundred-grain weight (g); Yield, yield per hectare (t/ha). **, significant at 0.01 level.

## Data Availability

The datasets generated during and/or analyzed during the current study are available from the corresponding author upon reasonable request.

## Supplementary Materials

The supporting information can be downloaded at: https://www.mdpi.com/article/10.3390/ijms232213943/s1.
