# Supplementary material for: Genetic Dissection of Phosphorus Use Efficiency and Genotype-by-Environment Interaction in Maize
Source: Int J Mol Sci. 2022 Nov 11;23(22):13943. doi: 10.3390/ijms232213943 (PMC9697416; doi:10.3390/ijms232213943)
Supplement: Supplementary file 1 [file ijms-23-13943-s001.zip › ijms-1990826-supplementary/all supplementary files_IJMS/Supplementary figures.pdf]

Supplementary Figures

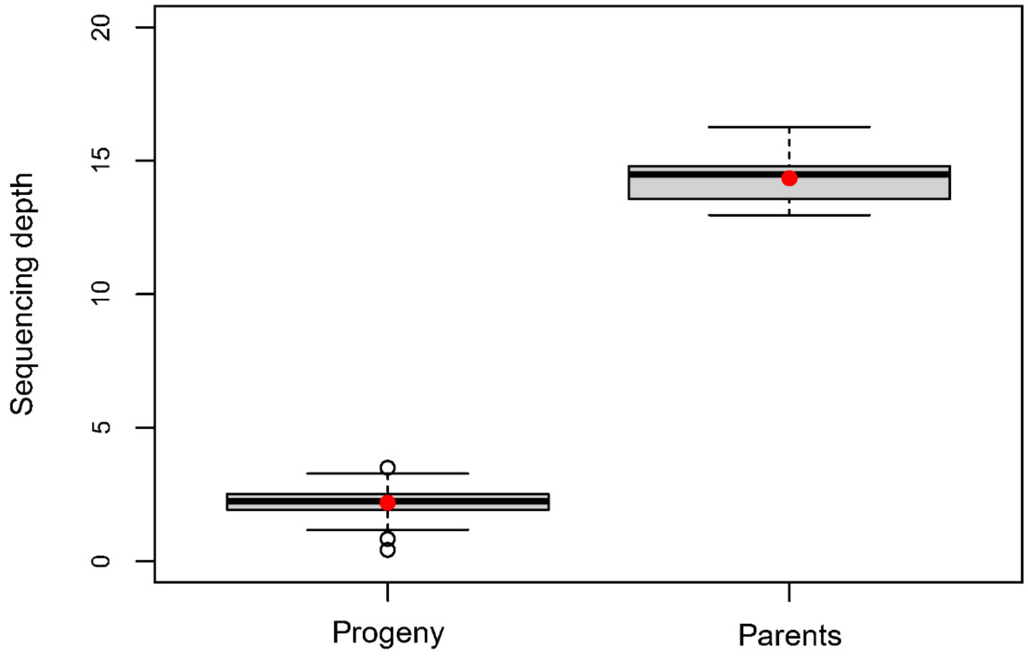

Figure S1. Sequencing depth of parents and progeny.

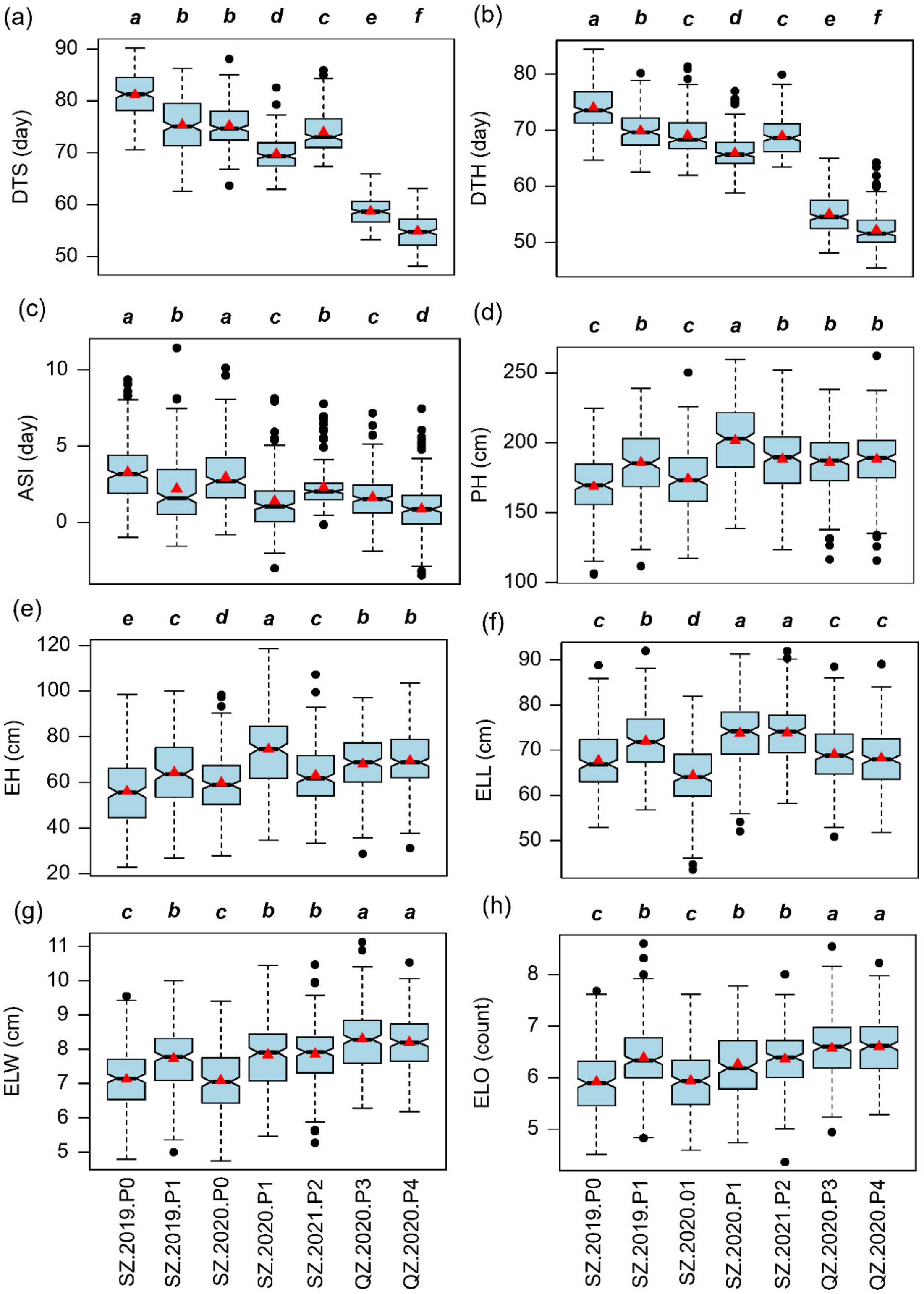
(a)
a b b d c e f
DTS (day)
90
80
70
60
50
(b)
a b c d c e f
DTH (day)
80
70
60
50
(c)
a b a c b c d
ASI (day)
10
5
0
(d)
c b c a b b b
PH (cm)
250
200
150
100
(e)
e c d a c b b
EH (cm)
120
100
80
60
40
20
(f)
c b d a a c c
ELL (cm)
90
80
70
60
50
(g)
c b c b b a a
ELW (cm)
11
10
9
8
7
6
5
(h)
c b c b b a a
ELO (count)
8
7
6
5
SZ.2019.P0
SZ.2019.P1
SZ.2020.P0
SZ.2020.P1
SZ.2021.P2
QZ.2020.P3
QZ.2020.P4
SZ.2019.P0
SZ.2019.P1
SZ.2020.01
SZ.2020.P1
SZ.2021.P2
QZ.2020.P3
QZ.2020.P4

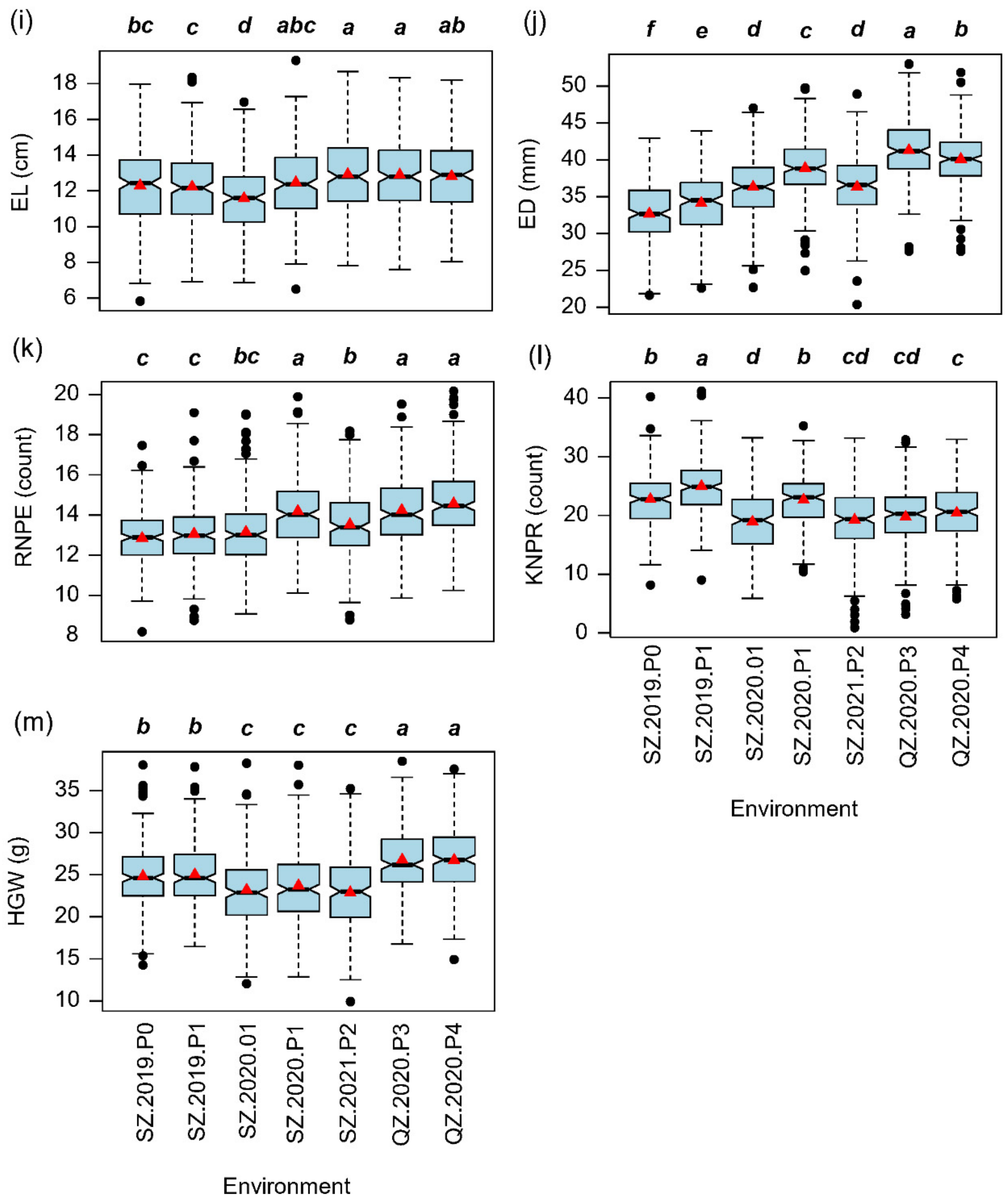

Figure S2. Phenotype distributions in the seven environments shown for 13 traits. (a) Days to silking (DTS). (b) Days to heading (DTH). (c) Anthesis-silking interval (ASI). (d) Plant height (PH). (e) Ear height (EH). (f) Ear leaf length (ELL). (g) Ear leaf width (ELW). (h) Ear leaf order (ELO). (i) Ear length (EL). (j) Ear diameter (ED). (k) Row number per ear (RNPE). (l) Kernel number per row (KNPR). (m) Hundred-grain weight (HGW). Multiple comparisons were done by the least significant difference method at the significance level of 0.05.

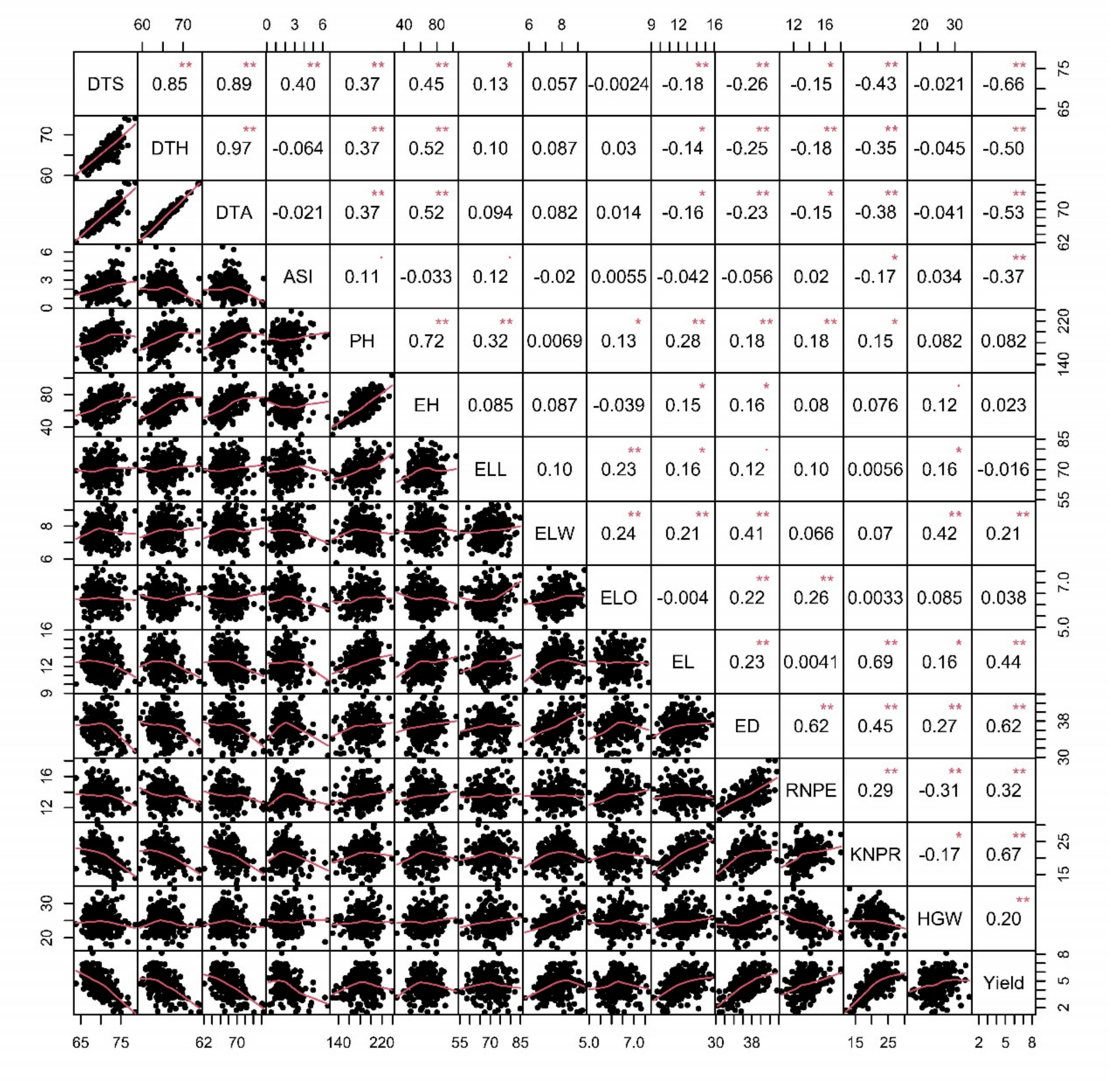

Figure S3. Correlations among the 15 traits. DTS, days to silking; DTH, days to heading; DTA, days to anthesis; ASI, anthesis-silking interval; PH, plant height; EH, ear height; ELL, ear leaf length; ELW, ear leaf width; ELO, ear leaf order; EL, ear length; ED, ear diameter; RNPE, row number per ear; KNPR, kernel number per row; HGW, hundred-grain weight; Yield, yield per hectare. *, significant at 0.05 level; **, significant at 0.01 level.

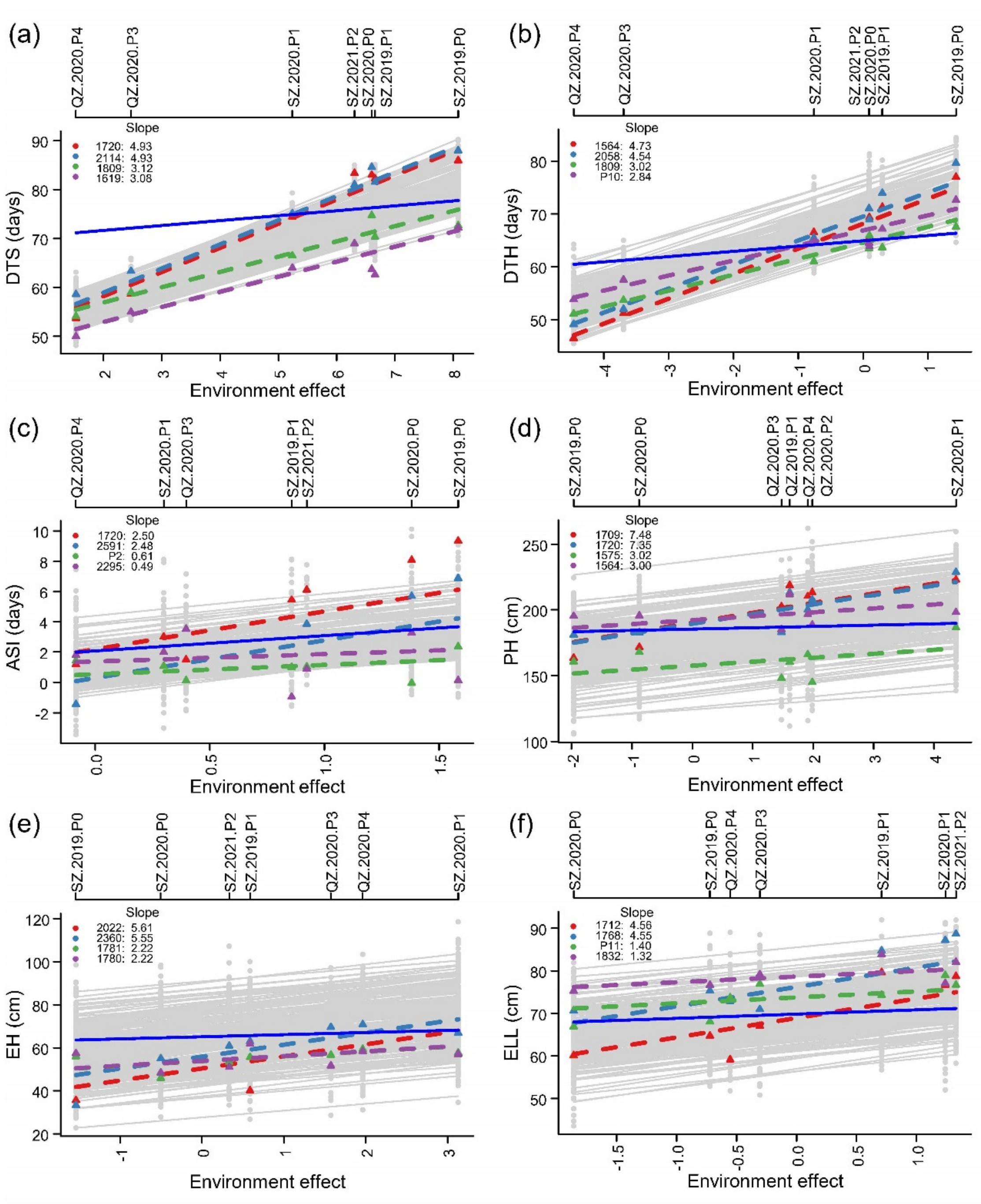
(a)
QZ.2020.P4
QZ.2020.P3
SZ.2020.P1
SZ.2021.P2
SZ.2020.P0
SZ.2019.P1
SZ.2019.P0
Slope
1720: 4.93
2114: 4.93
1809: 3.12
1619: 3.08
DTS (days)
Environment effect
(b)
QZ.2020.P4
QZ.2020.P3
SZ.2020.P1
SZ.2021.P2
SZ.2020.P0
SZ.2019.P1
SZ.2019.P0
Slope
1564: 4.73
2058: 4.54
1809: 3.02
P10: 2.84
DTH (days)
Environment effect
(c)
QZ.2020.P4
SZ.2020.P1
QZ.2020.P3
SZ.2019.P1
SZ.2021.P2
SZ.2020.P0
SZ.2019.P0
Slope
1720: 2.50
2591: 2.48
P2: 0.61
2295: 0.49
ASI (days)
Environment effect
(d)
SZ.2019.P0
SZ.2020.P0
QZ.2020.P3
SZ.2019.P1
QZ.2020.P4
SZ.2021.P2
SZ.2020.P1
Slope
1709: 7.48
1720: 7.35
1575: 3.02
1564: 3.00
PH (cm)
Environment effect
(e)
SZ.2019.P0
SZ.2020.P0
SZ.2021.P2
SZ.2019.P1
QZ.2020.P3
QZ.2020.P4
SZ.2020.P1
Slope
2022: 5.61
2360: 5.55
1781: 2.22
1780: 2.22
EH (cm)
Environment effect
(f)
SZ.2020.P0
SZ.2019.P0
QZ.2020.P4
QZ.2020.P3
SZ.2019.P1
SZ.2020.P1
SZ.2021.P2
Slope
1712: 4.56
1768: 4.55
P11: 1.40
1832: 1.32
ELL (cm)
Environment effect

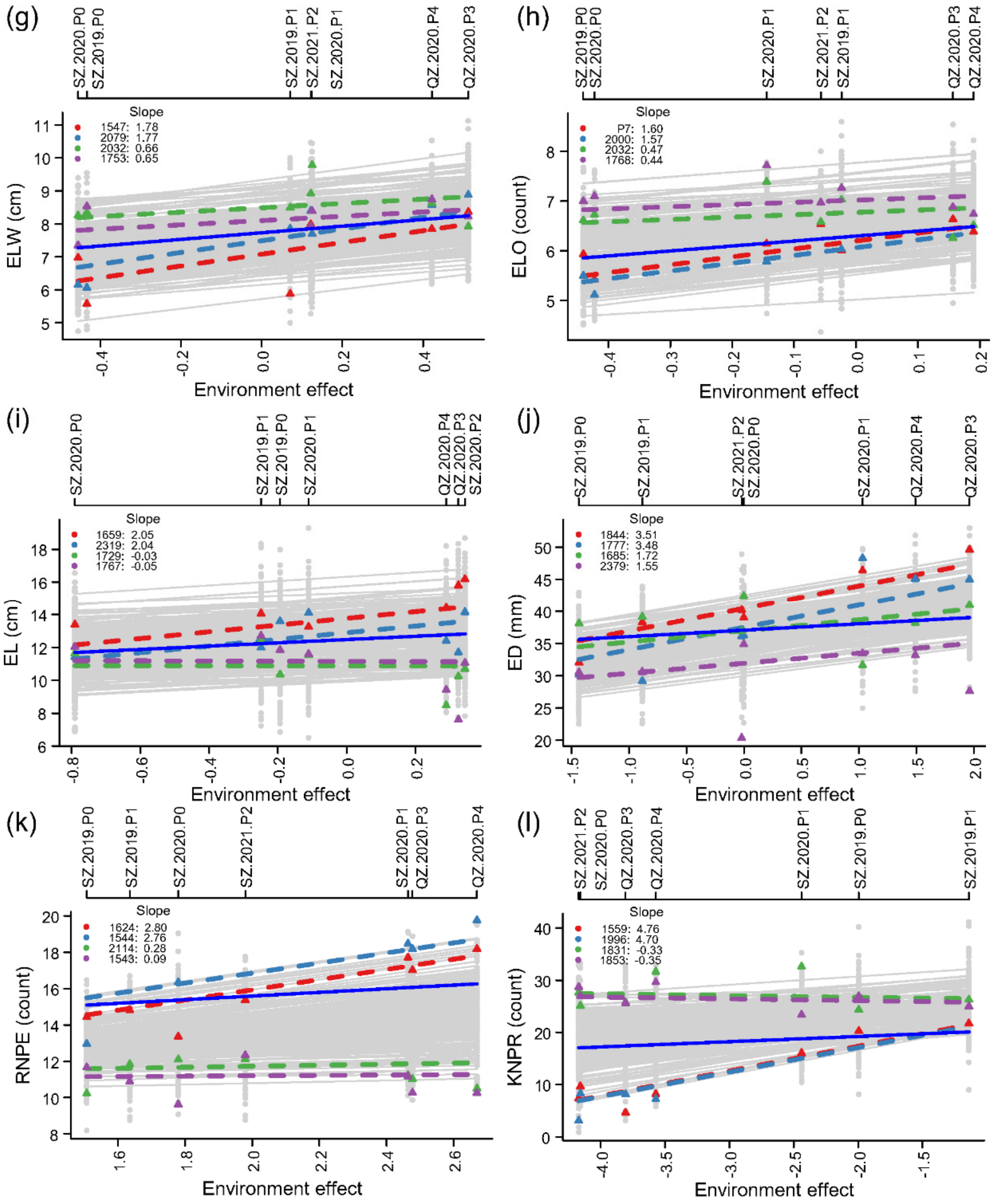
(g)
SZ.2020.P0
SZ.2019.P0
SZ.2019.P1
SZ.2021.P2
SZ.2020.P1
QZ.2020.P4
QZ.2020.P3
Slope
1547: 1.78
2079: 1.77
2032: 0.66
1753: 0.65
ELW (cm)
Environment effect
(h)
SZ.2019.P0
SZ.2020.P0
SZ.2020.P1
SZ.2021.P2
SZ.2019.P1
QZ.2020.P3
QZ.2020.P4
Slope
P7: 1.60
2000: 1.57
2032: 0.47
1768: 0.44
ELO (count)
Environment effect
(i)
SZ.2020.P0
SZ.2019.P1
SZ.2019.P0
SZ.2020.P1
QZ.2020.P4
QZ.2020.P3
SZ.2020.P2
Slope
1659: 2.05
2319: 2.04
1729: -0.03
1767: -0.05
EL (cm)
Environment effect
(j)
SZ.2019.P0
SZ.2019.P1
SZ.2021.P2
SZ.2020.P0
SZ.2020.P1
QZ.2020.P4
QZ.2020.P3
Slope
1844: 3.51
1777: 3.48
1685: 1.72
2379: 1.55
ED (mm)
Environment effect
(k)
SZ.2019.P0
SZ.2019.P1
SZ.2020.P0
SZ.2021.P2
SZ.2020.P1
QZ.2020.P3
QZ.2020.P4
Slope
1624: 2.80
1544: 2.76
2114: 0.28
1543: 0.09
RNPE (count)
Environment effect
(l)
SZ.2021.P2
SZ.2020.P0
QZ.2020.P3
QZ.2020.P4
SZ.2020.P1
SZ.2019.P0
SZ.2019.P1
Slope
1559: 4.76
1996: 4.70
1831: -0.33
1853: -0.35
KNPR (count)
Environment effect

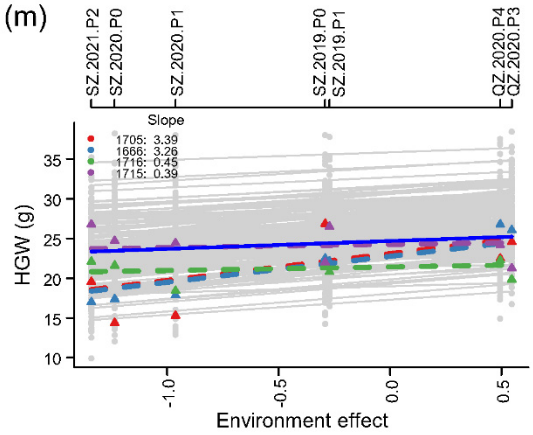

Figure S4. Finlay-Wilkinson regression analysis for (a) days to silking (DTS), (b) days to heading (DTH), (c) anthesis-silking interval (ASI), (d) plant height (PH), (e) ear height (EH), (f) ear leaf length (ELL), (g) ear leaf width (ELW), (h) ear leaf order (ELO), (i) ear length (EL), (j) ear diameter (ED), (k) row number per ear (RNPE), (l) kernel number per row (KNPR), and (m) hundred-grain weight (HGW). Only the four lines with the two highest and lowest linear plasticity for the trait are shown. The dashed lines represent the slope (linear plasticity). The greater the slope, the greater the linear plasticity of the plant. The solid blue line represents a slope of one.

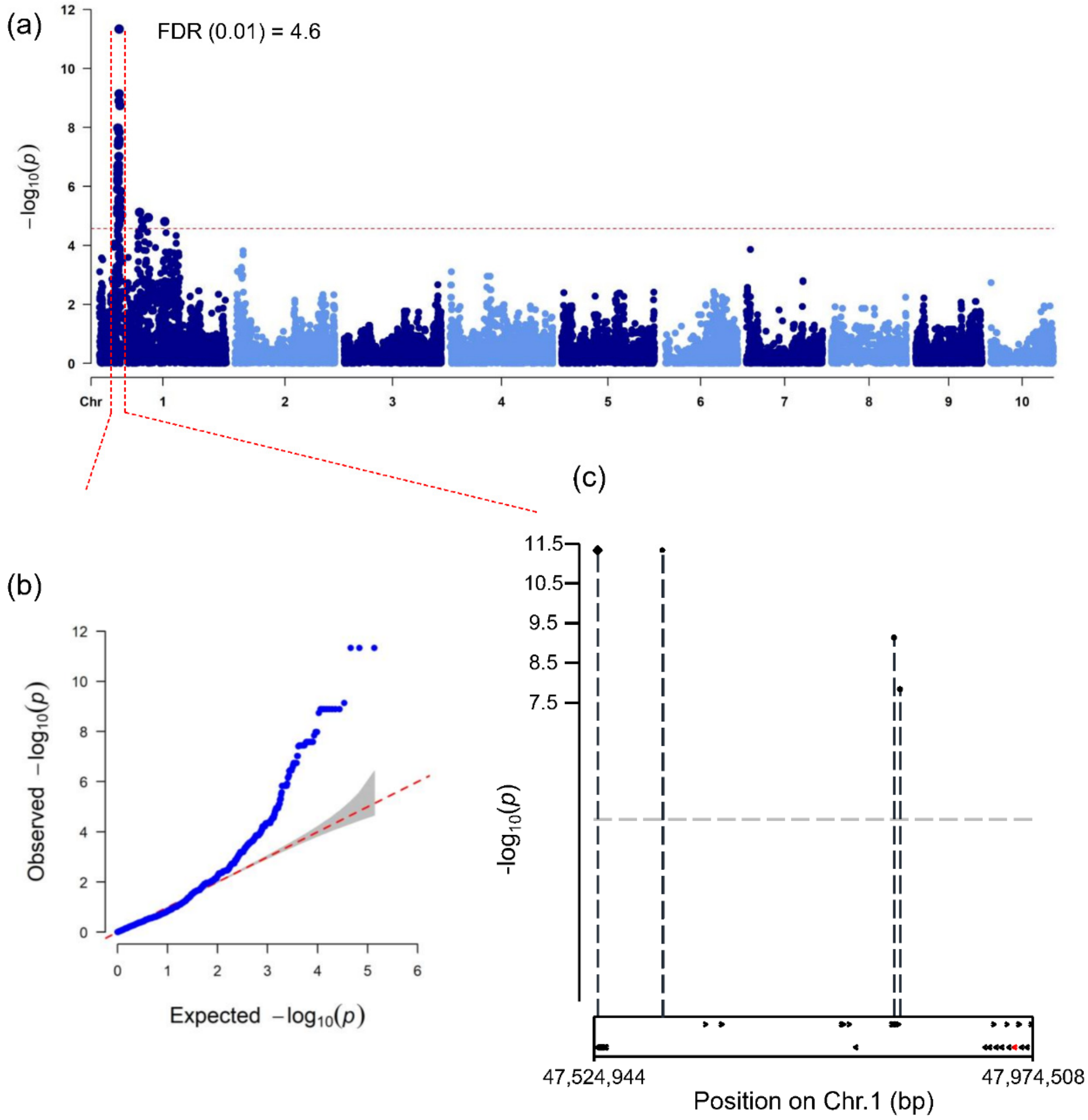

Figure S5. GWAS for cob color and identification of the gene *P1* (*pericarp color1*). (a) Genome-wide association mapping for cob color. (b) Quantile-Quantile plot for GWAS. (c) Identification of *P1* with a threshold of 4.6 determined by a false discovery rate (FDR) approach at the significance level of 0.01. The red arrow indicates the position of *P1*.
