# Supplementary material for: Genetic Dissection of Phosphorus Use Efficiency and Genotype-by-Environment Interaction in Maize
Source: Int J Mol Sci. 2022 Nov 11;23(22):13943. doi: 10.3390/ijms232213943 (PMC9697416; doi:10.3390/ijms232213943)
Supplement: Supplementary file 1 [file ijms-23-13943-s001.zip › ijms-1990826-supplementary/all supplementary files_IJMS/Supplementary tables.pdf]

Supplementary Tables

Table S1. Information of the maize population

	Source	Size	Pedigree	Female	Male
1	SYLO	17	BY/MC026	BY	MC026
2	HBYLZS	22	JIW134/JIST196	JIW134	JIST196
3	DDY	35	Dan3140/PHR58	Dan3140	PHR58
4	BJNL	38	Jing2481/LM	Jing2481	LM
5	HNND	44	L119A/Jing24	L119A	Jing24
6	SXDF	78	(F0147Z/A4190)/F0147Z	F0147Z	A4190
Sum		234			

Table S2. Dispersion and correlations between the linear and non-linear plasticity

Type	Trait	Quartile coefficient of dispersion	Correlation
Linear plasticity	DTS	0.06	-0.03
Non-linear plasticity		0.33	
Linear plasticity	DTH	0.06	0.02
Non-linear plasticity		0.46	
Linear plasticity	DTA	0.06	-0.09
Non-linear plasticity		0.42	
Linear plasticity	ASI	0.18	0.08
Non-linear plasticity		2.24	
Linear plasticity	PH	0.12	0.18**
Non-linear plasticity		0.10	
Linear plasticity	EH	0.14	0.18**
Non-linear plasticity		0.13	
Linear plasticity	ELL	0.13	0.22**
Non-linear plasticity		0.19	
Linear plasticity	ED	0.11	0.13
Non-linear plasticity		0.21	
Linear plasticity	EL	0.32	0.01
Non-linear plasticity		0.90	
Linear plasticity	ELW	0.18	0.08
Non-linear plasticity		-0.33	
Linear plasticity	ELO	0.24	-0.10
Non-linear plasticity		-0.19	
Linear plasticity	RNPE	0.23	0.13*
Non-linear plasticity		-5.66	
Linear plasticity	KNPR	0.40	0.28**
Non-linear plasticity		0.23	
Linear plasticity	HGW	0.28	0.04
Non-linear plasticity		0.31	
Linear plasticity	Yield	0.15	0.33**
Non-linear plasticity		0.88	

Note: Quartile coefficient of dispersion was calculated by (Q3-Q1) / (Q3+Q1), where Q1 and Q3 are 25-th percentile and 75-th percentile, respectively; **, significant at 0.01 level; *, significant at 0.05 level. DTS, days to silking; DTH, days to heading; DTA, days to anthesis; ASI, anthesis-silking interval; PH, plant height; EH, ear height; ELL, ear leaf length; ELW, ear leaf width; ELO, ear leaf order; EL, ear length; ED, ear diameter; RNPE, row number per ear; KNPR, kernel number per row; HGW, hundred-grain weight, Yield, yield per hectare
